# Canine Schistosomiasis in the West Coast: *Heterobilharzia americana* in Two Natural Intermediate Hosts Found in the Colorado River, California

**DOI:** 10.3390/pathogens13030245

**Published:** 2024-03-13

**Authors:** Anil Baniya, Connor J. Goldy, Jiranun Ardpairin, Perla Achi, Yu Wei Chang, Rose C. Adrianza, Apichat Vitta, Adler R. Dillman

**Affiliations:** 1 Department of Nematology, University of California, Riverside, CA 92521, USA; abaniya@ucr.edu (A.B.); cgold019@ucr.edu (C.J.G.); bachi001@ucr.edu (P.A.); yuwei.chang@email.ucr.edu (Y.W.C.); radri002@ucr.edu (R.C.A.); 2 Department of Microbiology and Parasitology, Faculty of Medical Science, Naresuan University, Phitsanulok 65000, Thailand; jiranuna61@nu.ac.th (J.A.); apichatv@nu.ac.th (A.V.); 3 Centre of Excellence for Biodiversity, Faculty of Sciences, Naresuan University, Phitsanulok 65000, Thailand

**Keywords:** range expansion, pathogen, schistosome, *Heterobilharzia americana*, *Galba cubensis*, *Galba humilis*, Colorado River, biodiversity, canine schistosomiasis

## Abstract

The emergence of infectious diseases presents a significant global health, economic, and security risk. Climate change can unexpectedly lead to the spread of pathogens, vectors, or hosts into new areas, contributing to the rise of infectious diseases. Surveillance plays a crucial role in monitoring disease trends and implementing control strategies. In this study, we document the first discovery of *Heterobilharzia americana*, a parasitic schistosome of mammals and its intermediate hosts *Galba cubensis* and *Galba humilis* along the banks of the Colorado River in California. We conducted multiple samplings of snails from various locations in the region with a previous history of canine schistosomiasis. Nucleotide sequencing of the multiple regions of the snails’ and parasites’ DNA revealed the coexistence of *G. cubensis* and *G. humilis*, both infected with *H. americana*. Phylogenetic analyses further validate the presence of *H. americana* in California, suggesting a wider distribution than previously reported. Our findings have implications for public health, veterinary medicine, and biodiversity conservation, contributing to developing effective control strategies to prevent the spread of this emerging infectious disease.

## 1. Introduction

The rise of new and infectious diseases poses a substantial and increasing risk to global health, the worldwide economy, and international security [1]. Most emerging infectious diseases are zoonotic and originate from domesticated animals and wildlife [2,3]. Climate change resulting in vector and intermediate host range expansion may have widespread effects on the emergence of infectious diseases [4,5]. Examples of this include the outbreak of canine schistosomiasis in Utah [6] and the range expansions of various diseases, such as ticks and the meningeal worm *Parelaphostrongylus tenuis* [7,8,9]. Another potential source for the emergence of new diseases is the encounter of a particular pathogen or parasite with immune-compromised hosts. Examples include the recent infection of the nematode *Ophidascaris robertsi* in a woman’s brain and the horizontal transmission of cancerous tapeworm cells from *Hymenolepus nana* to a man’s lungs [10,11]. Disease and pathogen surveillance is a critical component of the health system because it provides timely information to monitor disease trends, evaluate health outcomes, and set parasite control and elimination strategies before they cause widespread disease. Here, we outline the discovery of a pathogenic schistosome infecting both wild and domestic mammals, along with its intermediate hosts, on the banks of the Colorado River in California.

**Figure 1:**
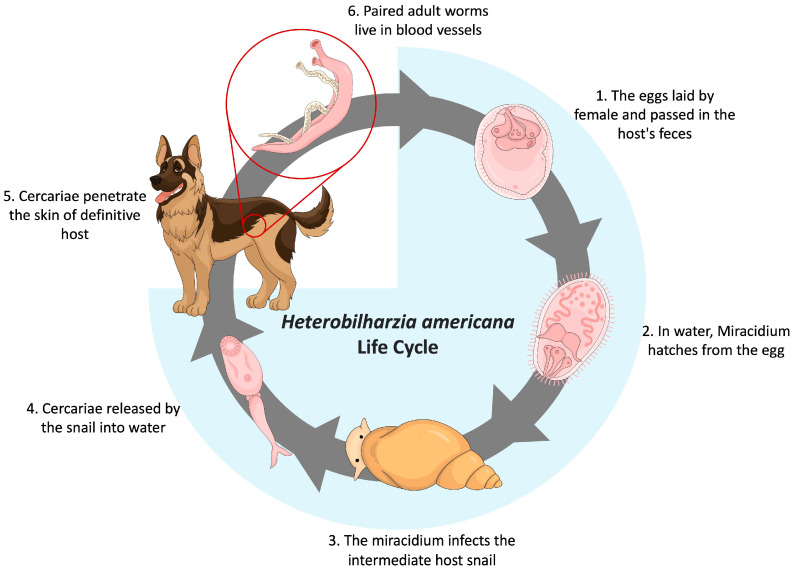
Life cycle of *Heterobilharzia americana*. Two-host life cycle contains six distinct stages: adult, egg, miracidium, sporocyst, cercaria, and schistosomula.

*Heterobilharzia americana* is a schistosome trematode endemic to the Gulf Coast and South Atlantic region of North America, infecting raccoons, marsh rabbits, dogs, horses, nutria, bobcats, mountain lions, opossums, and other mammals [12,13]. Nevertheless, there is a growing number of reports documenting the incidence of parasite infection across various new states, including Indiana [14], Tennessee [15], Oklahoma [13,16], Arkansas [13], Kansas [17], and, most recently, Utah [6]. The life cycle of *H. americana* is that of a typical digenic trematode within the family Schistosomatidae (Figure 1). Mammals that wade or swim in freshwater areas, such as marshes, mudflats, ponds, and canals, are exposed to *H. americana* [18]. The intermediate host snails release the free-swimming cercariae into the water, which then penetrate the definitive host’s skin. Once inside the skin, the parasite transforms into a juvenile form called a schistosomula. The young schistosomes first migrate to the lungs, where they can cause hemorrhaging [19]. Afterward, they reach the liver and undergo development into both male and female. The male and female migrate to the mesenteric veins, mate, and lay spine-free eggs. Eggs make their way into venules, penetrating the intestinal wall, and are then released into the intestinal lumen. They are eventually excreted in feces. Upon reaching freshwater, the eggs hatch rapidly, releasing ciliated miracidia that swim to locate a suitable snail host. In infected snails, asexual development leads to the formation of both mother and daughter sporocysts and cercariae which then migrate to the hepatopancreas/digestive gland. It takes approximately 68 days post-infection to observe eggs in the feces of the definitive host [15,19].

The schistosome *H. americana* has been reported to infect a wide range of mammalian species in various regions of the United States. However, for a long time, the only snail species believed to be a natural intermediate host for *H. americana* was the small amphibious lymnaeid, *Galba cubensis* [20]. In 2021, a new species of snail host, *Galba humilis*, was recorded as testing positive for natural infection with this pathogen [6], and in 2023, another snail species, *Galba schirazensis*, was reported to be a compatible intermediate host of *H. americana* [21]. Similarly, *Pseudosuccinea columella* [22], a widely distributed aquatic lymnaeid, is experimentally susceptible [23], but it has not been observed with natural infection. In the United States, *Galba cubensis* has been recorded in Alabama, California, Florida, Georgia, Louisiana, Mississippi, North Carolina, New Mexico, South Carolina, and Texas [24]. The historical range of *H. americana* appears to match the occurrence of this snail in most of these states. Populations of *G. cubensis* have also been recorded from South America, Mexico, the West Indies, and Europe [25,26]. Another intermediate host, *Galba humilis*, also has a wide geographical distribution within the USA from Alaska to Florida and worldwide including Canada, Japan, and South America [6,20,27,28,29,30].

Dogs, when exposed to *H. americana*, manifest symptoms such as dermatitis (caused by penetrating cercariae), vomiting, coughing, fever, bloody diarrhea, anorexia, weight loss, lethargy, polyuria, and polydipsia, ultimately leading to collapse. Granulomatous reactions provoked by *H. americana* eggs are notable in organs including the lungs, liver, pancreas, and intestine. Common clinicopathologic observations in infected dogs include anemia, dehydration, hyperglobulinemia, hypoalbuminemia, hypercalcemia linked to elevated parathyroid hormone-related protein levels, and eosinophilia [15,31,32,33,34]. Similar symptoms are observed in *H. americana*-infected raccoons and horses [35,36]. In dogs, *H. americana* infections are treated with high-dose praziquantel or fenbendazole, with variable outcomes ranging from complete cure to treatment ineffectiveness [32]. The prolonged nature of the disease, poor treatment response, and diagnostic challenges often lead to euthanasia, and the clinical signs associated with schistosomiasis can mimic those of tumors and other diseases, requiring expensive and invasive procedures that may delay definitive diagnosis and appropriate therapy [31,34]. Veterinarians consistently emphasize that *H. americana* is frequently overlooked in diagnoses and is becoming a growing concern, particularly in dogs, horses, and other mammals [13,14,15,16,37,38].

The discovery of *H. americana* and its natural intermediate hosts in the Colorado River is noteworthy. Understanding the pathogen’s dissemination should prompt preemptive control strategies. This becomes particularly crucial in averting substantial damage to dog populations and other natural mammalian communities. In the subsequent section, we explore the discovery of *H. americana* within its natural hosts, *Galba cubensis* and *Galba humilis*, coexisting along the banks of the Colorado River in California. This discovery serves as compelling evidence of westward expansion of *H. americana* in the United States.

## 2. Materials and Methods

### 2.1. Collection and Screening of Snails

Snails were collected from the banks of the Colorado River multiple times. The first sampling of the snail population was conducted on 1 March 2023, among 2 sites (Mayflower County Park (33.67119, −114.53319) and Quechan Park (33.60725, −114.53104)). The second sampling of the snails took place on 4 April 2023, with samples collected from four different sites: First Leavy Park, Quechan Park, Mayflower County Park, and Hidden Beaches (33.6566, −114.51738). The third sampling was carried out on 16 July 2023, specifically from the 2 sites (Quechan Park, and Hidden Beaches) where many snails were found during the second sampling. The collection of snails involved using handled kitchen strainers, which were swept through aquatic vegetation near the shoreline, or snails outside of shorelines at or above the waterline were picked using hands. Snails were washed, isolated in individual wells of 24-well plates, and left overnight in the dark before screening to identify individual snails shedding cercariae. Snails were re-screened the next morning. Snails that did not shed cercariae were maintained in the laboratory and re-evaluated every day for two weeks. Snails releasing cercariae were photographed, and both the individual snails and their cercariae were subjected to molecular identification.

### 2.2. DNA Extraction for Molecular Identification of Snails and Cercariae

Snail populations from individual surveys were grouped based on shell morphology, and DNA extraction was performed on each morphotype for molecular identification. Total DNA was extracted from the foot tissue of the snails. Each snail was placed under the microscope, and a small piece of foot tissue was cut using a sharp scalpel. The tissue was then placed in 30 μL of extraction buffer in a 200 µL microcentrifuge polymerase chain reaction (PCR) polypropylene tube, mixed with 27 μL of 10 mM Tris, 1 mM EDTA pH 8.0 (Thermo Fisher Scientific, Fair Lawn, NJ, USA), 1.5 μL of 2% Triton X, and 1.5 μL of Proteinase K (20 mg/mL, New England Biolabs, Ipswich, MA, USA). The snail tissue was disrupted by freezing and thawing five times using liquid nitrogen, and then the tissue was incubated overnight at −20 °C. The following day, the frozen lysate was incubated at 56 °C for 1 h, followed by 95 °C for 10 min. DNA from cercariae was extracted using a similar technique as described above. Approximately 100 cercariae were placed in 20 μL of extraction buffer in a 200 µL microcentrifuge PCR tube, mixed with 18 μL of 10 mM Tris, 1 mM EDTA pH 8.0 (Thermo Fisher Scientific, Fair Lawn, NJ, USA), 1 μL of 2% Triton X, and 1 μL of Proteinase K (20 mg/mL, New England Biolabs, Ipswich, MA, USA). A similar protocol was followed to disrupt the cell and extract DNA as described for snail tissues above.

### 2.3. DNA Amplification and Sequencing

Amplification of multiple loci in snail and cercariae DNA was performed using different primer sets. We used 1 µL of the DNA extract as a template for a 25 µL PCR reaction, using 12.5 µL of Taq polymerase (Promega, Madison, WI, USA), 1 µL each of forward and reverse primer, and 9.5 µL of nuclease-free for all the PCR tests. Snail ITS1 was amplified using Lim1657 (forward) 5′-CTGCCCTTTGTACACACCG-3′, ITS1-RIXO (reverse) 5′-TGGCTGCGTTCTTCATCG-3′ [20]. Snail ITS2 was amplified using NEWS2 (forward) 5′-TGTGTCGATGAAGAACGCAG-3′ and ITS2-RIXO (reverse) 5′-TTCTATGCTTAAATTCAGGGG-3′ [39]. Thermocycling conditions for snail ITS1 (Lim1657, ITS1RIXO) were as follows: 94 °C for 6 min; 3 cycles for each annealing temperature, 60–56 °C then 20 cycles at 55 °C with denaturation at 94 °C for 30 s and extension at 72 °C for 2 min, and a final extension at 72 °C for 7 min. Similarly, the PCR conditions for ITS2 consisted of an initial denaturation step at 94 °C for 2 min. This was succeeded by 32 cycles, with intervals of 30 s at 94 °C, 30 s at 50–58 °C, and 30 s at 72 °C. The process concluded with a final extension step of 7 min at 72 °C. The COX 1 gene of the snails was amplified using LCOI490 (forward) 5′-GGTCAACAAATCATAAAGATATTGG-3′ and HCO2198 (reverse) 5′-TAAACTTCAGGGTGACCAAAAAATCA-3′ [40]. The PCR condition for the amplification of the COX I gene was denaturation at 94 °C for 2 min, 30 cycles of 30 s at 94 °C, 30 s at 52 °C, and 2 min at 72 °C, followed by a final 7 min extension at 72 °C.

For the parasite DNA, amplification of mitochondrial cytochrome oxidase subunit 1 (CO1) was conducted using Cox1_schist_5 (forward) 5′-TCTTTRGATCATAAGCG-3′, Cox1_schist_3 (reverse) 5′-TAATGCATMGGAAAAAAACA-3′ [41] and CO1F15 (forward) 5′-TTTNTYTCTTTRGATCATAAGC-3′, and CO1R15 (reverse) 5′-TGAGCWAYHACAAAYCAHGTATC-3′ [42]. The amplification of the CO1 gene used the same PCR conditions as described above for the amplification of the CO1 gene in the snails. Amplification of the large subunit region was performed using the primers C1 (forward) 5′-ACCCGCTGAATTTAAGCAT-3′ and D2 (reverse) 5′-TGGTCCGTGTTTCAAGAC-3′ [43].

PCR results were visualized on 1% agarose gel stained with 0.0003% ethidium bromide, along with a 1 kb plus DNA ladder (New England Biolabs, Beverly, MA, USA). Gel purification of the PCR products was performed using the QIAquick^®^ Gel Purification Kit (Qiagen, Germantown, MD, USA), following the manufacturer’s protocol. Subsequently, the purified PCR products were sent for Sanger sequencing using both the forward and reverse strands at the UCR Core Instrumentation Facility, according to the manufacturer’s protocol. The chromatogram sequence uncertainties in both the forward and reverse sequences for each locus underwent visual inspection and assembly through SeqManII software (DNASTAR Inc., Madison, WI, USA). The sequences generated as part of this work have been deposited in NCBI GenBank with their corresponding accession for snail and *H. americana* sequences (Supplementary Materials).

### 2.4. Identification and Phylogenetic Analysis

Identification of closely related species for each amplicon from snails and *Heterobilharzia americana* was conducted using the Basic Local Alignment Search Tool (BLAST) within the National Center for Biotechnology Information (NCBI) database. Based on the blast result, different closely related species of snails and *H. americana* species were used to construct the phylogeny. All sequences were aligned using MAFFT [44] with default parameters and the aligned sequences were visualized using Clustal W in MEGA 1; any alignment inconsistencies were corrected manually [45]. The IQ-Tree program [46] was used to choose the best model utilizing the ‘find best model’ function and to construct a maximum likelihood (ML) phylogenetic tree, incorporating ultrafast bootstrap branch support with 1000 replicates.

## 3. Results

### 3.1. Survey Results

**Figure 2:**
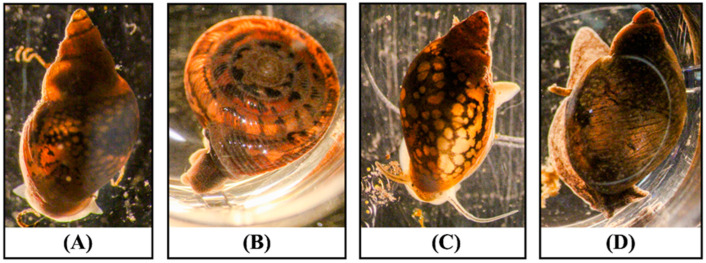
Different morphologies of snails collected during the snail survey. (**A**) Right-handed cone-shaped snail (*Galba* spp.). (**B**) Spiral-shaped snails (*Polygyra* spp.). (**C**) Left-handed cone-shaped snails with long tentacles (*Physella acuta*). (**D**) Big cone-shaped snails (*Succinea* spp.).

From the first sampling, we collected 524 snails. Those snails were divided into four different groups based on the shell morphology (Figure 2). For all our surveys, the first and predominant category (comprising more than 90%) of snails were right-handed cone-shaped snails with very high shell spires, round and unobtrusive tentacles, and a foot that was yellowish to light olive. The second group of snails had spiral whorls, giving the shell a uniform wood-brown or fawn-colored appearance, with the last whorl swollen at the end. The third group of snails was composed of left-handed cone-shaped snails with a size like that of the snails in the first group but with longer tentacles. The fourth group of snails had larger shell sizes compared to all the snails collected during our survey, with a large, inflated body whorl and a short shell. During our second and third surveys, we collected 664 and 788 snails, respectively. Since the only recorded hosts of *H. americana* are cone-shaped snails, our focus was solely on this type of snail, and we exclusively gathered specimens of this kind.

For the second survey, we extracted DNA from four randomly selected snails at each of the three locations (Hidden Beach, First Leavy Park, and Mayflower County Park) in group A. For group B snails, we randomly selected four individuals. In group C, all four left-handed snails collected during the survey underwent DNA extraction. Additionally, three snails were selected from group D. The amplification and sequencing of the cytochrome oxidase subunit I (COX1) genes generated fragments ranging in size from 594 to 680 base pairs (bp). The Basic Local Alignment Search Tool (BLAST) analysis of the sequenced genes revealed that the majority of snails from group A were identified as two snail species, *Galba humilis* and *Galba cubensis*, with identity ranging from 98.76% to 100%. All the snails from group B were identified as *Polygyra* spp., with identities ranging from 96.74% to 99.83%. Snails from group C were identified as *Physella acuta*, with identities ranging from 98.23% to 99.56%. Similarly, snails from group D were identified as *Succinea* spp., with identities ranging from 99.50% to 99.83% (Supplementary Materials).

**Figure 3:**
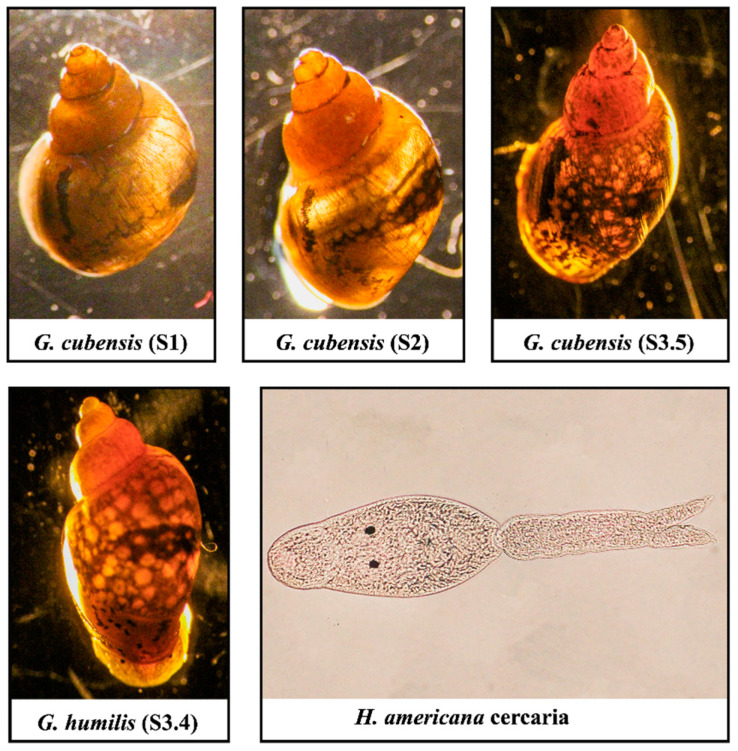
Images of three *Galba cubensis* from the top and one *Galba humilis* from the bottom that shed cercariae. The cercaria of *H. americana* had two eyespots, a tail, and swam freely as a morphological feature on the lower left panel. The codes S1, S2, S3.4, and S3.5 represent individual isolates of snails that were shedding cercariae.

During our third survey, we focused solely on collecting cone-shaped snails (*Galba* spp.) from group A, with the majority obtained from Hidden Beach (33.6566, −114.51738). We collected 788 snails, and among them, four were shedding schistosome cercariae. These four snails underwent DNA extraction and sequencing (Figure 3 and Supplemental Videos). The sequencing of the COX I gene produced sequences ranging in size from 486 to 642 bp. BLAST analysis identified three snails as *Galba cubensis*, with identities ranging from 98.14% to 98.68%. Another fragment, measuring 655 bp in length from the fourth snail, was identified as *Galba humilis* with an identity of 99.52% in the database. We performed DNA extraction from four cercariae, amplifying the partial large subunit (28S) and cytochrome oxidase subunit I (COX1) gene. The 28S fragment lengths ranged from 897 bp to 902 bp, and the COX I gene fragment lengths ranged from 463 bp to 955 bp. BLAST results of the 28S sequences showed high similarity to the *Heterobilharzia americana* Utah strain, with a percent identity ranging from 99.56% to 99.89%. Similarly, BLAST results of the COX I gene for the California cercariae were highly similar to the *H. americana* Utah strain, with a percent identity ranging from 97.69% to 99.17%.

### 3.2. Phylogenetic Analysis of the Snail

**Figure 4:**
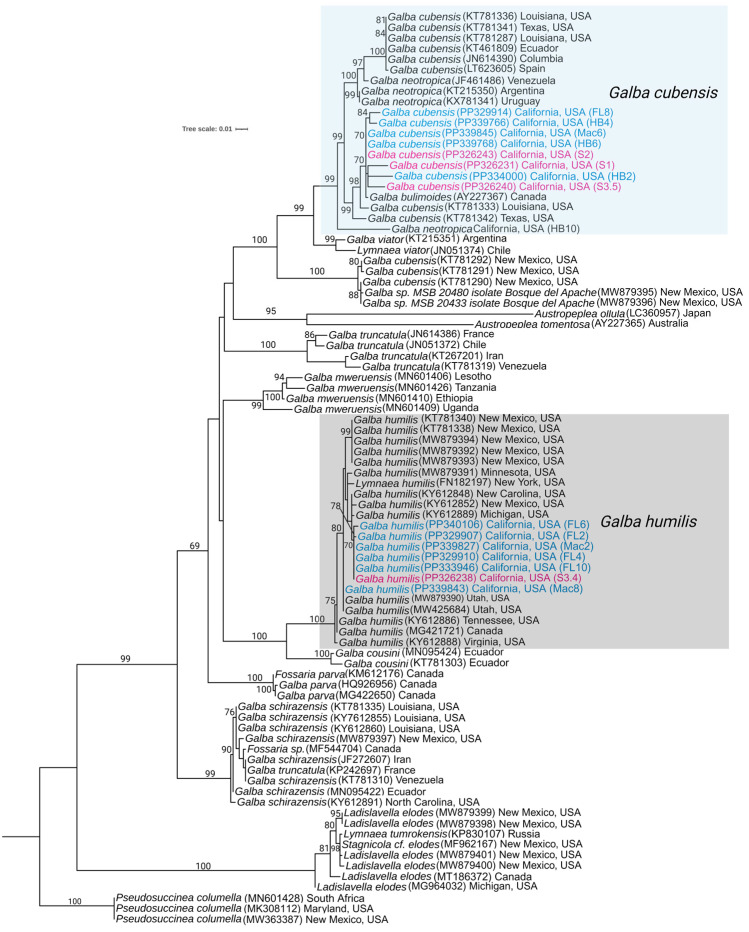
Maximum likelihood phylogenetic trees of snails collected during the snail survey based on cytochrome oxidase subunit I (COI1). The analysis was run with a bootstrap value of 1000. Numbers at the branches represent bootstrap support values for each node. The snails collected during the second sampling are marked in blue, while those from the third sampling that were positive for cercariae infection are indicated in pink. The clade representing *G. humilis* is delineated by a gray box, while *G. cubensis* and other species are indicated by light blue boxes. The names of taxa are presented with their corresponding GenBank accession number preceding them and their collection locality (US state) and other parts of the world following them.

For the phylogenetic analysis involving the cytochrome oxidase subunit I (COX1) gene of snails, we incorporated sequence data from both the second and third surveys. In the second survey, we included snails from three locations, selecting 4 snails from each site, resulting in a total of 12 snail samples. In the third survey, we specifically considered the sequence information from four snails that were shedding cercariae. Utilizing the BLAST results from NCBI for the sequenced snails, we identified 60 distinct snail isolates for comparison in constructing the phylogeny. In total, 86 sequences from different species were used for sequence alignment using MAFFT, and the IQ-Tree program determined K3Pu+F+I+G4 as the most fitting model for constructing a maximum likelihood (ML) phylogenetic tree. In the COX1 phylogeny, *Pseudosuccinea columella* from South Africa, Maryland, and New Mexico, with accession numbers MN601428, MK308112, and MW233387, respectively, were employed as an outgroup (Figure 4).

Based on the COX1 phylogeny, all the lymnaeid from group A were divided into two clades: *Galba cubensis* and *Galba humilis*. Species of snails in the *Galba cubensis* clade collected from California are strongly supported, as they clustered with representatives of this species from geographically distant regions within the United States, Canada, and South America. Similarly, *Galba humilis* species collected during our study group with strong statistical support, aligning with other snail species collected in the United States, such as Utah, New Mexico, and North Carolina. This robust support for the two clades suggests that these freshwater snails coexist in the same habitat along the banks of the Colorado River in California (Figure 4).

**Figure 5:**
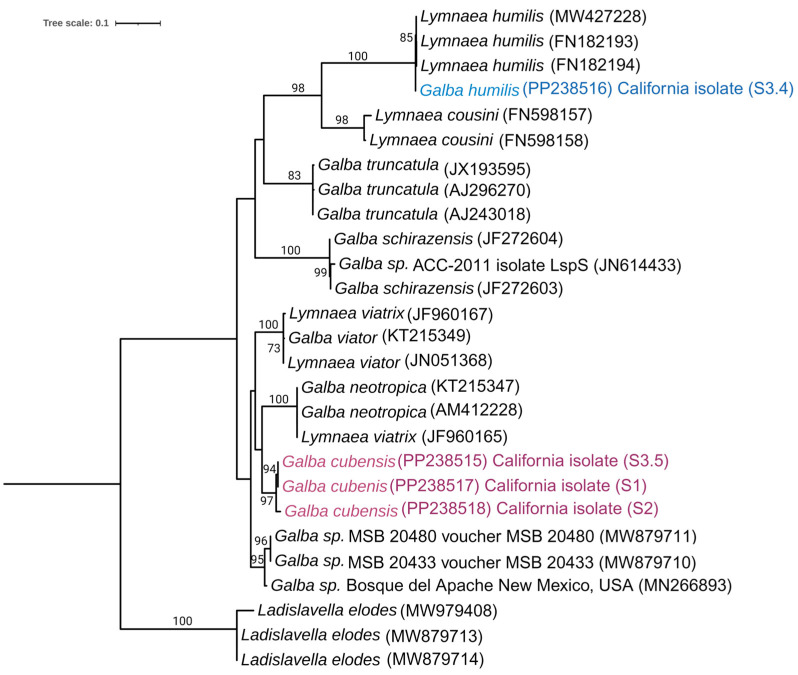
Maximum likelihood phylogenetic tree of *Galba* species based on ITS1 sequences recovered during our survey that were shedding *Heterobilharzia americana* cercariae. Values on the nodes indicate bootstrap branch support with 1000 replicates. The isolate of *G. humilis* recovered in our study is delineated in light blue and the isolates of *G. cubensis* are delineated in light pink.

In another phylogenetic analysis of snails, we sequenced the partial Internal Transcribed Spacer 1 (ITS1) genes of four snails that were shedding cercariae. By utilizing the BLAST results from NCBI for these sequenced snails, we identified 23 distinct snail isolates for comparison in constructing the phylogeny. In total, 27 sequences from different species were used for sequence alignment using Mega 11, and the IQ-Tree program determined TPM2+G4 as the most fitting model for constructing a maximum likelihood (ML) phylogenetic tree based on the Bayesian Information Criterion (BIC). Different isolates of *Ladislavella elodes*, with accession numbers MW979408, MW879713, and MW879714 were employed as an outgroup (Figure 5).

**Figure 6:**
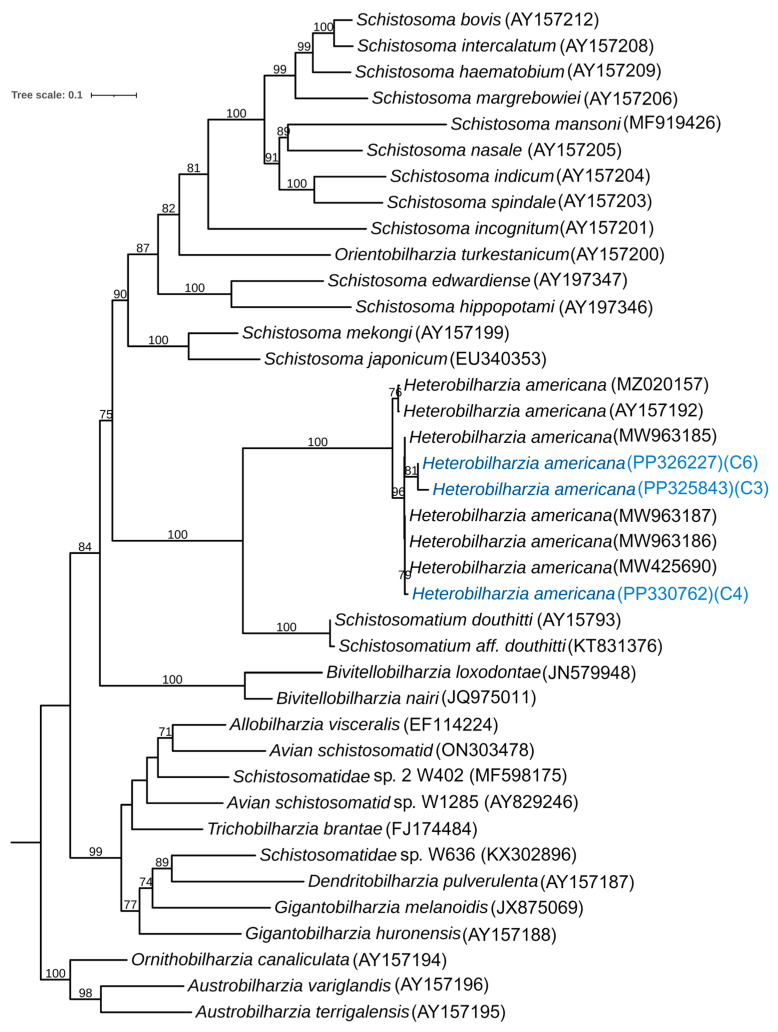
Maximum likelihood phylogenetic trees for relevant schistosomes, including *Heterobilharzia americana* identified from naturally infected snails in California, based on cytochrome oxidase subunit I (COX1) genes. Nodal support values are bootstrap branch support with 1000 replicates. The *H. americana* strain indicated in blue represents the samples collected during our study. The corresponding GenBank accession numbers of each isolate are listed after taxa names.

A phylogenetic study of H. americana was conducted using the cytochrome oxidase subunit I (COX1) and the 28S region of the large subunit. For the phylogenetic tree based on COX1, we sequenced three isolates of cercariae and, based on the BLAST results in NCBI, identified 36 different isolates for the phylogenetic study. Based on the alignment of 39 sequences, the IQ-Tree program determined GTR+F+I+I+R4 as the best fitting model for constructing a maximum likelihood (ML) phylogenetic tree, based on the Bayesian Information Criterion (BIC) (Figure 6). The determination of the outgroup was conducted based on the previous analyses [6].

*Heterobilharzia americana* cercariae collected during our study clustered together and were placed next to *H. americana* collected in Utah. Furthermore, all the strains of *H. americana* were clustered together in the same clade next to *Schistosomatium douthitii*, suggesting close relationships between *H. americana* and *S. douthitti*. The results also revealed that isolates of *H. americana* from California are more similar to those from Utah and Texas than to an isolate from Louisiana (Figure 6).

**Figure 7:**
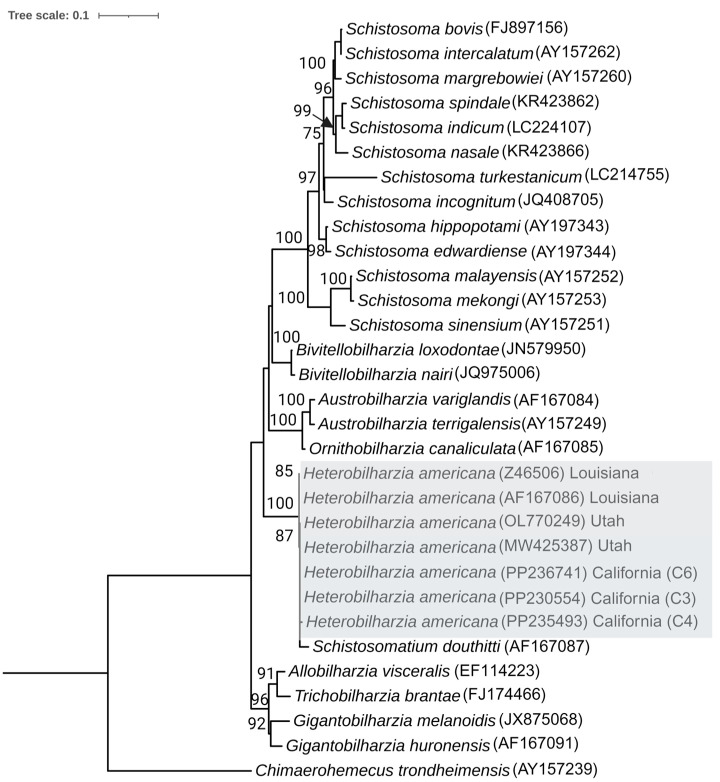
Maximum likelihood estimates of the interrelationships of the Schistosomatidae based on the 28S sequences. Nodal support values are bootstrap branch support with 1000 replicates. The clade belonging to *H. americana* strain collected during our study and its close relative strain are indicated in the gray box. The corresponding GenBank accession numbers of each isolate are listed after taxa names.

Sequencing of the 28S region of *H. americana* and BLAST analysis on GeneBank resulted in the identification of 28 different species of Schistosomatidae. A total of 31 sequences were aligned for the study of their interrelationship. The IQ-Tree program determined TVM+F+G4 as the most fitting model for constructing a maximum likelihood (ML) phylogenetic tree based on the Bayesian Information Criterion (BIC). The outgroup was determined as *Chimaerohemecus trondheimensis* based on a previous study [41]. All the isolates of the *H. americana* were placed together in the same clade next to *Schistosomatium douthitii*, confirming their close relationship and validating the maximum likelihood tree based on the COX1 gene (Figure 7).

## 4. Discussion

In 2023, the Los Angeles County of Public Health issued an advisory confirming 11 cases of canine schistosomiasis in dogs from three Southern California counties: Los Angeles, Orange, and Riverside, between 2018 and 2023. The parasite had not previously been reported as endemic to Southern California. Travel history of the infected dogs indicated that before their diagnosis, all 11 dogs had been in the Colorado River [37]. This finding led us to conduct our survey [12,14,17,18,22,31,32,35]. Based on our study, we were able to recover *H. americana* cercariae from two naturally occurring snails, *Galba cubensis* and *Galba humilis*, coexisting together along the banks of the Colorado River. This finding helps to support the notion that the infected dogs likely contracted *H. americana* from the Colorado River. This finding is also the most westerly report of this endemic North American schistosome, suggesting that this parasite has a wider range than previously speculated and is expanding its distribution beyond what was previously reported [6].

**Figure 8:**
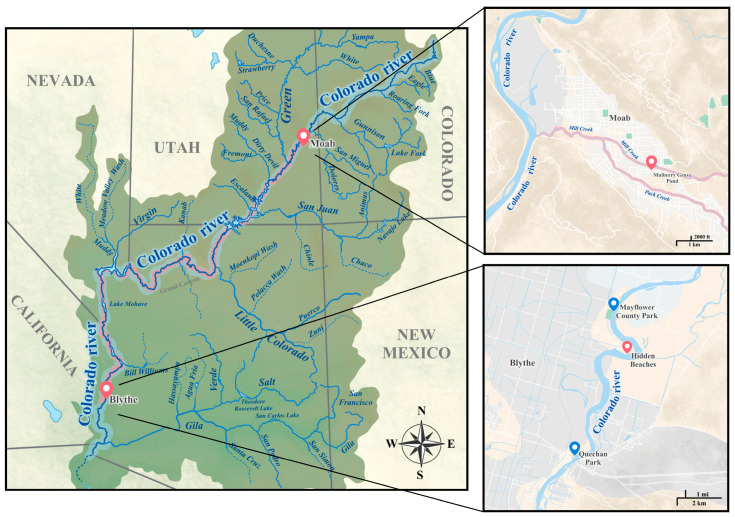
A detailed map of the Colorado River and its tributaries in the United States, depicting various locations of the presence of *Heterobilharzia americana* and its natural hosts. The top extended map illustrates the first recorded instance of *H. americana* in its natural host, *Galba humilis*, in Moab, Utah. The extended map at the bottom shows the sampling locations in California where the two natural hosts, *Galba humilis* and *Galba cubensis*, were recorded coexisting together infected with *H. americana*. Locations where *H. americana* was reported is marked in red. Blue indicates locations where snails were collected but *H. americana* infection in snails was not confirmed.

Initially, *G. cubensis* was exclusively considered to be the natural host of *H. americana* [18,20]. Another species, *G. humilis* from Michigan, showed marginal susceptibility upon experimental exposure to *H. americana* [12]. However, in 2021, *G. humilis* was discovered to be naturally infected with *H. americana* in a human-made pond in Moab, Utah. This discovery was recorded at Mulberry Grove Pond, which receives water from Mill Creek, one of the tributaries of the Colorado River. This pond is approximately 280 feet from Mill Creek, and the shore of the Colorado River is approximately 2.17 miles away from the pond [6]. The newly identified natural host had a 77.5% positive infection rate following experimental exposure of *H. americana*, suggesting increased compatibility with *G. humilis*, which is widely distributed across North America [6]. Recently, another snail species *Galba schirazensis* was newly reported to be a compatible host of *H. americana* [21]. Our study provides the first report of the coexistence of two snail species, *G. cubensis* and *H. humilis*, in the same location during multiple samplings, both naturally infected with *H. americana*. This validates a wider distribution of *H. americana* than previously speculated (Figure 6). *Galba* is a genus of small-shelled freshwater snails that primarily originated in the Americas and subsequently invaded Asia, Africa, and Europe [47,48,49,50,51]. Their capacity to endure periods of drought and reproduce through self-fertilization enables them to spread across extensive distances and establish new populations from individual snails [47,52]. Their high invasiveness may be one reason for the broader expansion of the parasite associated with these snails [53]. Our phylogenetic analysis, which revealed other *Galba* species closely related to *G. cubensis* and *G. humilis*, combined with the identification of diverse snail species in our survey, indicates the potential for other snail species to be potential hosts of *H. americana*. Our finding of *H. americana* on the shore of the Colorado River in the southernmost part of the United States leads us to hypothesize that the spread of this parasite is throughout the Colorado River and among its tributaries. This is because the Colorado River and its watershed area harbor a diversity of mammals that can serve as hosts for this parasite, contributing to the maintenance of *H. americana* populations [54]. In-depth studies are required to investigate the extent of parasite infestation along the banks of the Colorado River and its tributaries in the United States and Mexico to gain a clear understanding of the parasite’s infection (Figure 8).

Our primary sampling site is located on the banks of the Colorado River in a popular spot for recreation. The area is also abundant in vegetation, attracting various mammals such as raccoons, bobcats, and rabbits. The identification of *H. americana* from this location suggests that these naturally occurring mammals may be infected with this parasite, contributing to its population maintenance. The extent of infection in the natural mammal population in this area remains unknown. Further investigation is needed to understand the impact of this parasite on our biodiversity, as it poses a potentially severe threat. A survey in Texas emphasized the severity of *H. americana* infection among raccoons, indicating a prevalence of up to 47% in raccoons of all ages and up to 85% in older raccoons [55]. This high prevalence was suspected to be the cause of *H. americana* infection in horses in the same locality [36].

The study of *H. americana* biology presents a unique challenge due to limited information about the identification and distribution of the intermediate host, coupled with scant details about the schistosome itself. *Schistosoma*, a genus of blood flukes, is responsible for causing schistosomiasis—a widespread and challenging disease affecting both humans and animals. Approximately 200 million people are infected with schistosomes, and an estimated 500–600 million individuals in various tropical and subtropical countries are at risk of contracting the disease [56]. Furthermore, the penetration of cercariae from animal schistosomes into human skin can result in irritating outbreaks of cercarial dermatitis, commonly known as swimmer’s itch [57]. There are at least 13 identified genera and more than 100 species of schistosomes to date. The larval stage of these schistosomes infects intermediate host snails and parasitizes a variety of organisms, including birds and mammals [58,59].

The schistosome identified in our survey, *H. americana*, is primarily a parasite of domestic and wild mammals. The exact host range of this parasite is not known; further studies are required to validate the susceptibility range. A study conducted in 1967, where *H. americana* was experimentally introduced into humans, revealed that the parasite caused vesicles at the infection site, and the rash subsided 5–8 days postinfection. In infected rhesus monkeys, no eggs were recovered from the feces; however, young schistosomules were retrieved from the liver 21 days after exposure, and pre-adults were observed in the liver of a second monkey 45 days after exposure [22]. Another study revealed a variable range of susceptibility among primates [60]. There is much more to explore regarding the pathogenicity and host range of this parasite, highlighting the need for further experimentation.

## 5. Conclusions

In our study, we successfully confirmed the presence of *Heterobilharzia americana* for the first time along the shores of the Colorado River, infecting two species of snails, *Galba humilis* and *Galba cubensis*. This significant finding marks the westernmost record of this endemic North American schistosome in the United States. The identification of the parasite in an area with a documented history of canine schistosomiasis emphasizes the persistence and potential expansion of this parasitic threat. This parasite’s presence in the area suggests a broader distribution than previously reported, highlighting the need for a better understanding of its geographical range. Such information is critical given the potential impact on natural biodiversity, domestic pets, and human health. The threat posed by *H. americana*, with its complex life, warrants careful consideration and proactive measures. Future research should focus on understanding the severity of the infestation, the dynamics of the parasite’s life cycle, and its interactions with various hosts. An in-depth understanding of these aspects is crucial for formulating effective strategies to manage and control the spread of *H. americana*. To conclude, our study sheds light on the expanding range of *H. americana*. These findings underscore the need for continued research, surveillance, and strategic planning to mitigate the potential ecological, veterinary, and public health impact of this parasite.

## Data Availability

The sequence reads generated in this study have been deposited in the NCBI Sequence Read Archive (SRA) database with individual accession numbers. Details about the individual snails and cercariae used in this study are provided in Supplementary Tables S1 and S2. Images of all the snails used for DNA extraction during the second survey are provided in Supplementary Figure S1. A video of the cercariae released from the snails is included in the Supplementary Videos.

## Supplementary Materials

The following supporting information can be downloaded at: https://www.mdpi.com/article/10.3390/pathogens13030245/s1, Figure S1: Images of *Galba cubensis* and *Galba humilis* collected during our second survey that were used for DNA extraction and successive identification; Tables: A detailed table delineating taxonomic information for each DNA sample extracted during our work from snails and *Heterobilharzia americana*. Table S1: Datasheet containing all information on sequenced snail DNA samples. Table S2: Data sheet with all information about *Heterobilharzia americana* DNA sequences; Videos: Videos S1 and S2 freely swimming *Heterobilharzia americana* released from individual snails collected during the third survey.
